# Implantation of Novel Meniscus Scaffold for Irreparable Meniscal Tear

**DOI:** 10.1016/j.eats.2021.12.036

**Published:** 2022-04-22

**Authors:** Shuhei Otsuki, Kuniaki Ikeda, Kei Tanaka, Yoshinori Okamoto, Shunsuke Sezaki, Masashi Neo

**Affiliations:** a Department of Orthopedic Surgery, Osaka Medical and Pharmaceutical University, Japan; b QOL Research Laboratory, Gunze Limited, Kyoto, Japan

## Abstract

Partial meniscectomy, which is generally used for the treatment of meniscal tears, can lead to knee joint osteoarthritis. To prevent this important complication, attempting to restore normal knee joint kinematics and biomechanical forces after partial meniscectomy is essential. Implantation of a meniscal scaffold can be useful in this regard, improving the function of the meniscus on knee joint biomechanics after partial meniscectomy. Use of meniscal scaffolds would have specific clinical merit for young patients who are at highest for developing knee joint osteoarthritis over time. Herein, we describe our novel bioabsorbable meniscal scaffold, fabricated with polyglycolic acid coated with polylactic acid/caprolactone, used after partial meniscectomy for degenerative and irreparable meniscal tears. The method of implantation of the scaffold will have a determinant effect on clinical outcomes. As the implementation technique by arthroscopy will be influenced by the stiffness and strength of the scaffold implant used, we provide a detailed description of our implantation technique, including a description of the pitfalls to consider in order to improve clinical outcomes.

## Introduction

The meniscus plays a key role in maintaining the homeostatic environment of the knee joint by facilitating force transmission, shock absorption, joint stability, and lubrication, as well as proprioception. Owing to the loss of meniscal function after tearing and the consequent negative impact of this loss of function on knee joint biomechanics and health, several surgical treatments have been developed for the management of meniscal tears, including suturing, partial or total meniscectomy, implantation of artificial meniscus,^1^ allogeneic meniscus,^2^ and implantation of a meniscal scaffold.^.^^3^^,^^4^ Of these options, suturing and excision of the degenerative meniscus are the main treatment approaches used. However, suturing is associated with a 15-30% risk of rerupture,^5^ while resection is associated with a 14-fold higher risk of knee joint osteoarthritis (OA),^6^ as it decreases the contact area and increases contact pressure between adjacent surfaces of the knee joint.^7^ Meniscal allograft transplantation can address the limitations of meniscal suturing and excision.

The long-term outcomes after viable meniscal allograft transplantation are encouraging in terms of pain relief and knee joint function, with improvement in function of >80% having been reported over a 10-year follow-up period.^2^ However, the use of allografts raises concerns regarding immunogenicity and infection. Moreover, access to allografts is limited in several countries due to social contexts, such as religion and beliefs around the use of human donors. Meniscal scaffold implants provide a viable alternative to the use of allografts.

**Fig 1:**
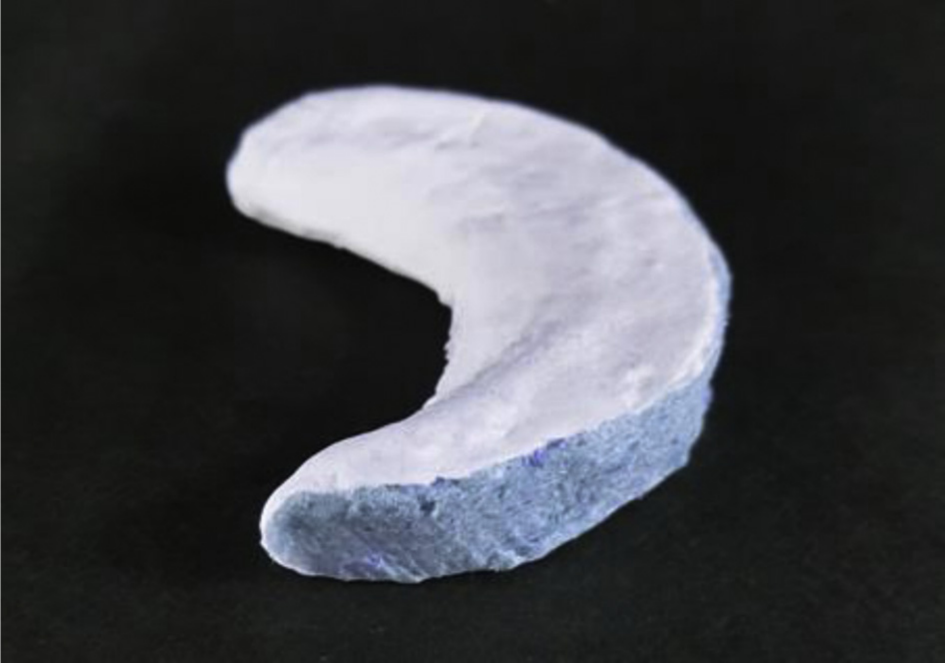
Meniscal scaffold composed of PGA coated with polylactic acid/caprolactone or P(LA/CL).

The goal of using bioabsorbable meniscal scaffolds is to preserve the biomechanical function of the native menisci by regenerating its size. Although scaffolds do not yield long-term regeneration of functional meniscal tissue or prevent OA,^8^ they would have specific short-term clinical merit after partial meniscectomy for young patients who are at highest risk for developing knee joint OA due to prolonged changes in knee joint biomechanics. In a previous study, we have shown that polyglycolic acid (PGA) is a useful material for tissue regeneration of the meniscus with the positive effects of a novel meniscus scaffold composed of PGA coated with polylactic acid/caprolactone or P(LA/CL) having been demonstrated in a mini pig model (Fig. 1).^9^ In the clinical context, however, outcomes are influenced by the surgical technique used for arthroscopic implantation of the PGA scaffold. Therefore, the purpose of this technical note is to describe our arthroscopic technique for implantation of our PGA scaffold after partial meniscectomy performed for treatment of degenerative and irreparable meniscal tears. We include a description of the pitfalls of the technique to consider improvement in clinical outcomes.

### IRB and Informed Consent

Ethics approval for the clinical trial was granted by the institutional review board of Osaka Medical and Pharmaceutical University (no. 21-3-08-0474), and all patients provided written informed consent before enrollment.

## Surgical Technique

**Fig 2:**
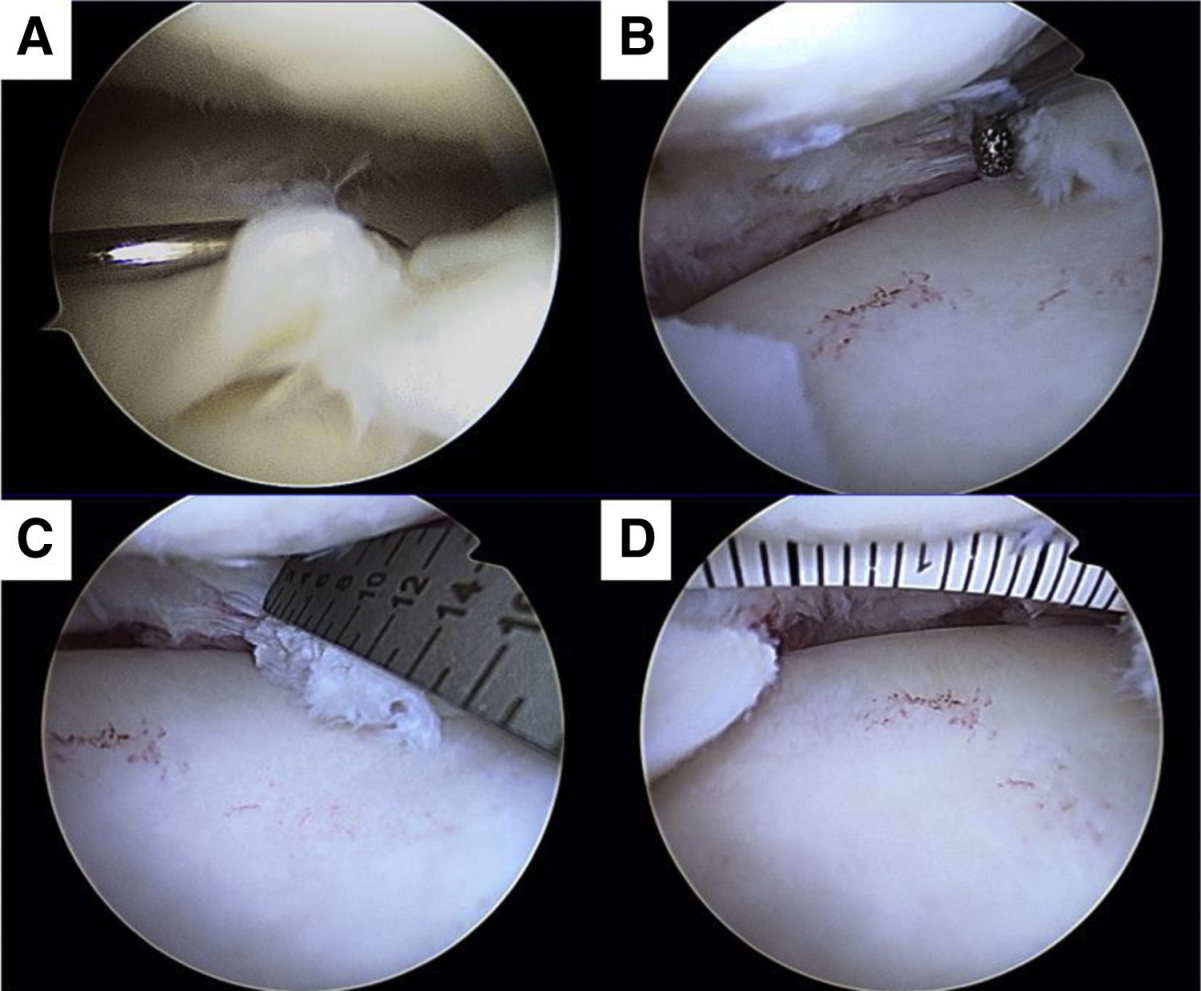
(A) Arthroscopic view from anterolateral portal showed degenerative flap tear with horizontal at posterior medial meniscus. (B) Rasping was performed after partial meniscectomy. (C, D) Measurement of the size of meniscal defect. After partial meniscectomy, defect area was measured to set up the meniscal scaffold.

Our surgical technique is presented in Video 1. An arthroscopic view from the anterolateral portal is performed for diagnostic evaluation of the meniscus to confirm presence of a horizontal degenerative flap tear of the posterior medial meniscus (Fig 2A). Partial meniscectomy is performed to remove the degenerative meniscus, followed by rasping (Fig 2B). The size of the meniscal defect in the case presented is within the acceptable range for scaffold implantation: width, 16 mm and depth, 14 mm (Fig 2, C and D).

### Indications

Implantation of a PGA scaffold is indicated after partial meniscectomy for a degenerative meniscal tear that cannot be repaired by suturing, in individuals between the ages of 18 and 60. The peripheral rim of the meniscus must be preserved, and the size of the defect must be <1/3 of the length of the circumference, as a large defect area might induce instability of the implanted scaffold. Lastly, the hip–knee–ankle angle should be within ±3° of the neutral angle to ensure a normal coronal knee alignment. The preoperative exclusion criteria include predisposition to allergic symptoms, such as bronchial asthma and urticaria; obesity, defined as a body mass index (BMI) ≥30 kg/m^2^; disruption of the cruciate ligaments, resulting in knee joint instability; and surgery of the target knee joint, such as ligament repair and osteotomy, within the previous 6 months. The intraoperative exclusion criteria are a cartilage damage >1 cm^2^ and an International Cartilage Repair Society (ICRS) classification grade ≥III.

### Segmental Meniscal Scaffold Preparation

**Fig 3:**
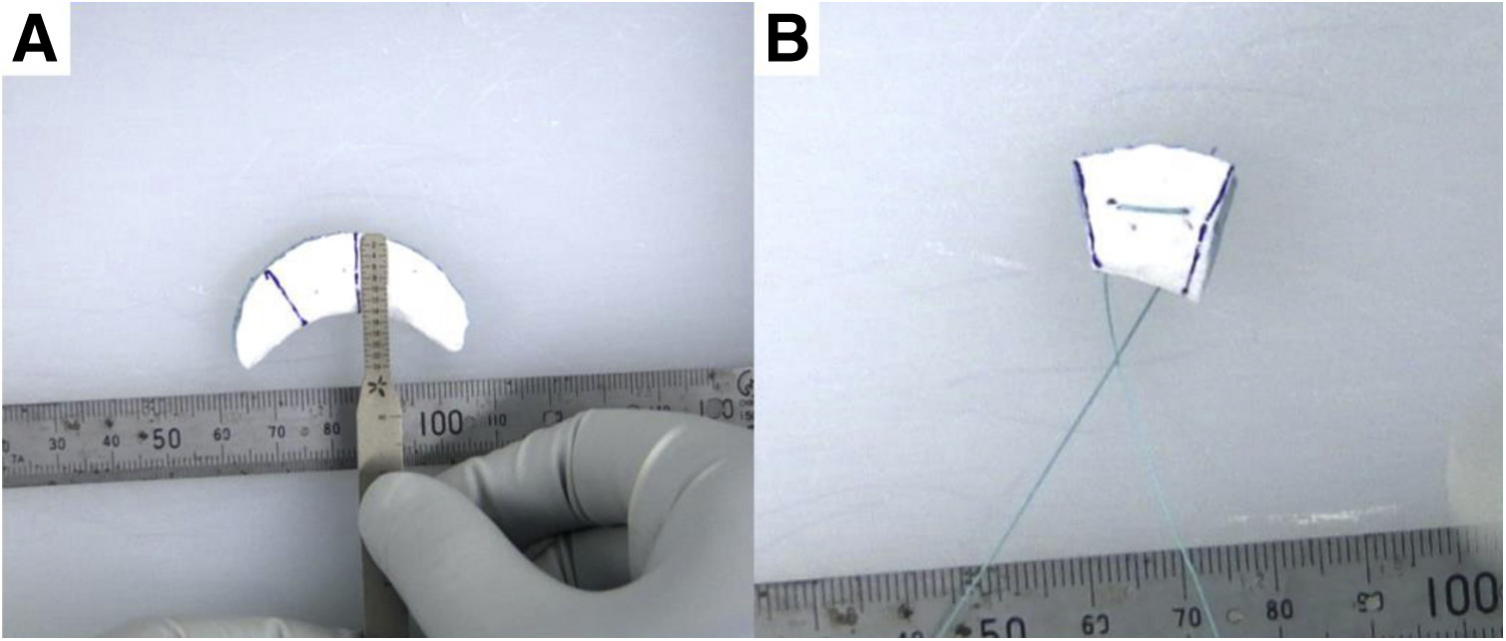
(A) Meniscal scaffold was set up the implantation size. (B) To control the scaffold during arthroscopy, the center of scaffold was set the rein suture.

The meniscal scaffold is trimmed to be 1-2 mm larger than the size of the defect area measured to ensure sufficient contact between the scaffold and the native meniscus (Fig 3A). To control the position of the scaffold during the arthroscopic procedure, a rein suture is set at the center of the scaffold (Fig 3B).

### Implantation of Meniscal Scaffold and Fix with Native Meniscus

**Fig 4:**
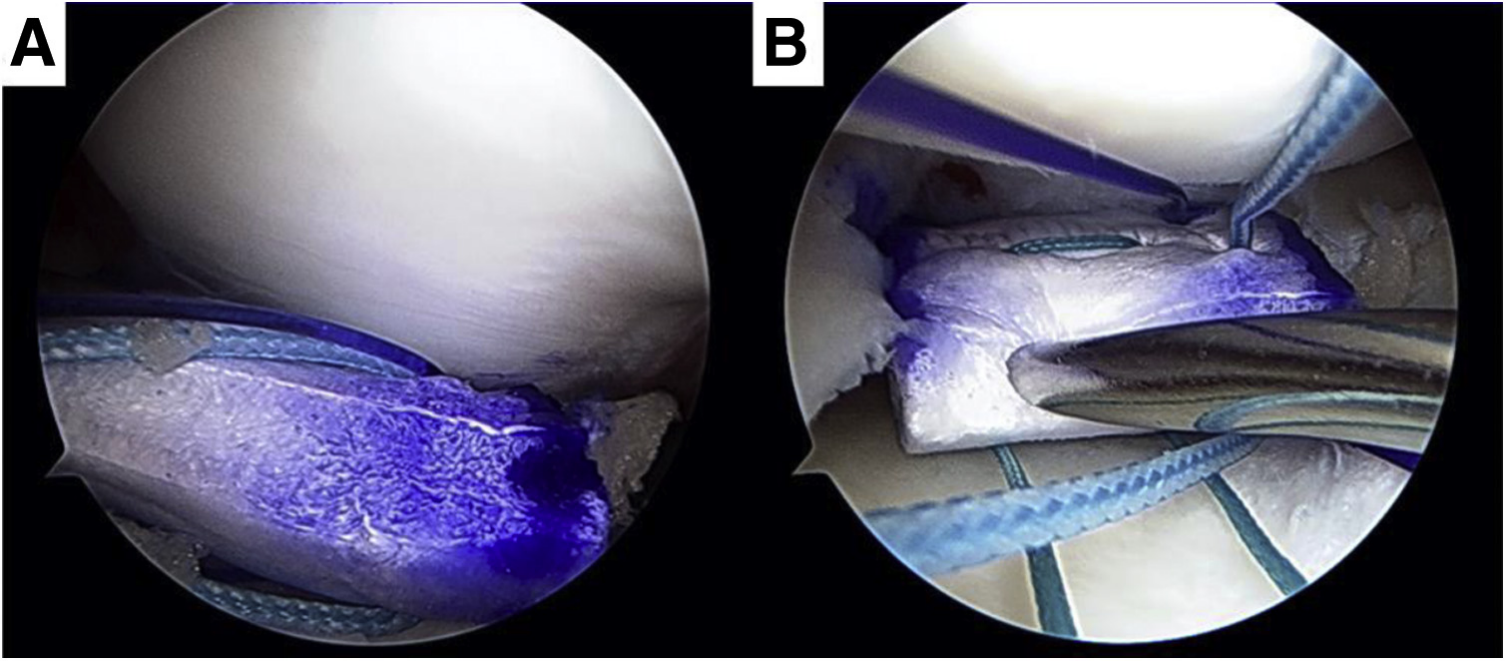
(A) Bring the meniscal scaffold in the knee joint from anteromedial portal. (B) Rein suture (green) was pulled so as not to push the peripheral outside by the inside-out suture needle.

**Fig 5:**
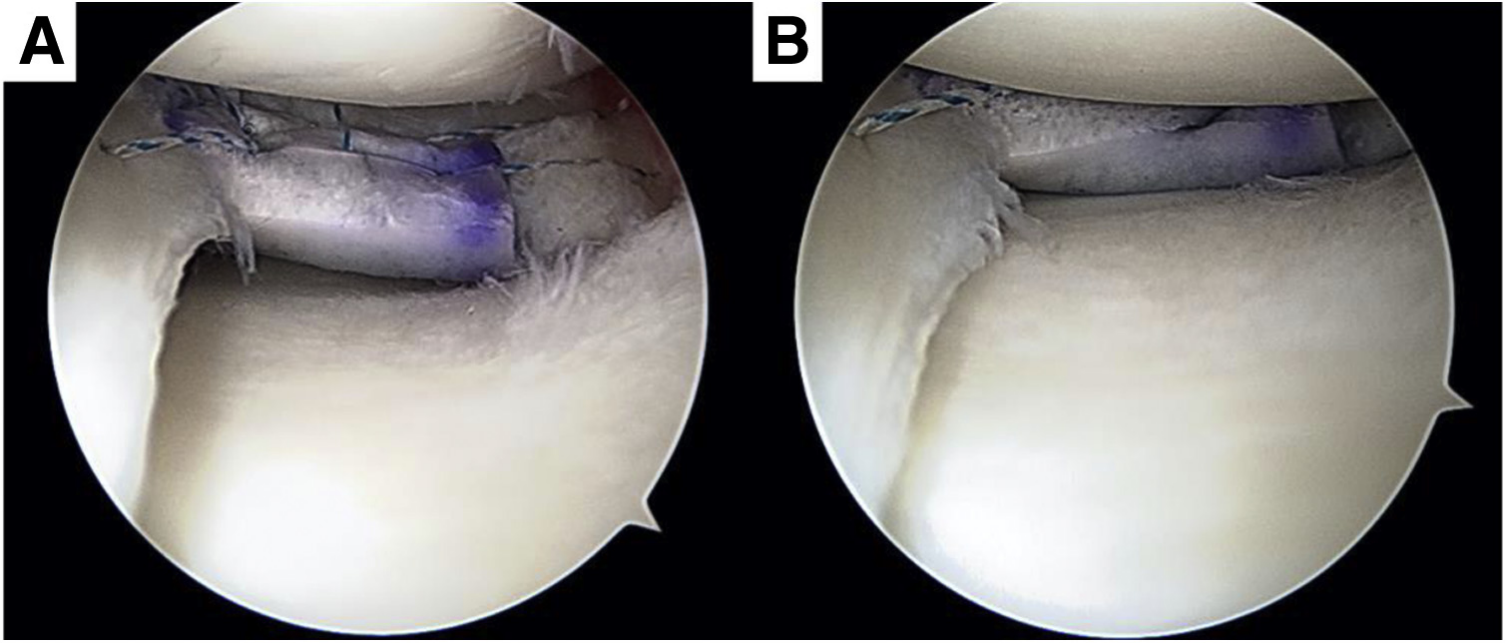
Implanted meniscal scaffold was fixed with inside-out or all-inside suture technique.

The synovium is removed via the anterior portal, allowing for implantation of the scaffold. The prepared meniscus scaffold is appropriately located on the meniscal defect using a grasper (Fig 4A). The rein suture is then pulled to avoid peripheral displacement of the scaffold during inside-out suture fixation of the scaffold to the native meniscus (Fig 4B). The inside-out suture technique is performed using a double-armed meniscus needle (CONMED, Utica, NY; Fig 5). The scaffold is first sutured to the native posterior horn and then to the central area of the meniscus, as recommended because of the relative lower flexibility of the posterior horn compared to the rest of the meniscus. As a result of this differential stiffness, gapping between the scaffold and the native menisci can easily occur during knee motion. The advantages and disadvantages, and pearls and pitfalls of the technique described are summarized in Tables 1 and 2, respectively.

**Table 1:** Advantages and Disadvantages of Meniscal Scaffold Implantation

Advantages	Disadvantages
Original meniscal size is preserved.	Indication of scaffold implantation is limited.
Restoration of biomechanical condition.	Peripheral meniscus must be preserved.
There is the potential to stop the progress of cartilage degeneration.	Fixation technique is complicated.
It provides access to the scaffold without immunogenicity and religious concerns	Long-term outcome has not been clarified.

**Table 2:** Pearls and Pitfalls of Meniscal Scaffold Implantation

Pearls	Pitfalls
To control the scaffold position, the rein suture should be set at the center of the scaffold before intra-articular translation.	Inside-out suture fixation makes it difficult to fix appropriate position because it tends to push the scaffold to the peripheral side.
Scaffold should be fixed at the posterior horn first because it is less flexible and easy to make the gap during knee motion.	Suture fixation should be having the posterior and the middle meniscus in turn.
The size of one portal is needed over 1cm, and soft tissue should be removed around the portal by shaver because the implanted scaffold is brought intraarticular smoothly. Using cannula might be another solution.	

### Postoperative Rehabilitation

Patients completed a postoperative program of rehabilitation as per the recommendations for meniscal scaffolds.^3^^,^^4^ Passive range of motion through full range of the knee joint is initiated on postoperative day 3, using a continuous passive motion device. Partial and full weight bearing are permitted at 4 and 8 weeks after surgery, respectively.

## Discussion

The main functions of the meniscus are shock absorption and joint stability, with the essential goal of meniscal repair being to reconstruct the hoop strength within the native size of the meniscus to preserve the kinematics and biomechanics of the knee joint. Meniscal allograft transplantation is a reasonable treatment for symptomatic meniscal deficiency. Fresh-frozen or deep-frozen grafts have commonly used, despite low cell viability, because of their relatively high success rates.^10^ The use of segmental meniscus allografts has also been recently reported after partial meniscectomy.^11^ Limitations of allografts, however, include insufficient recellularization of the native meniscus and inferior stiffness of the graft.^12^ Meniscal scaffolds provide an attractive alternative to allografts in terms of immunogenicity, infection, and effective reconstruction of the native size of the meniscus.

Meniscal scaffolds generally come in two varieties: collagen-based implant (collagen meniscal implant (CMI), Ivy Sports Medicine, Gräfelfing, Germany)^3^ and a polyurethane-based scaffold (ACTifit; Orteq, London, UK).^4^ The clinical outcomes for these two scaffolds have been evaluated, with the failure rate varying widely between 0 and 38% at 4 years postimplantation.^8^ The bioabsorbable PGA scaffold coated with P(LA/CL) that we developed improves the initial biomechanical strength of the meniscus.^9^ The surgical technique for scaffold implantation in clinical practice, however, is a critical aspect to consider, particularly as there is currently no report on the pitfalls of the technique used for scaffold implantation. On the basis of our experience, the PGA with P(LA/CL) coating scaffold is easy to position and to suture under arthroscopic technique. However, achieving the appropriate suture tension between the scaffold implant and the native menisci is somewhat difficult. Appropriate tension is important to avoid reinjury after fixation. To address this important limitation, we recommend the use of a rein suture, located at the center of the scaffold. After the scaffold is appropriately located in the knee joint, the rein suture is pulled to avoid peripheral displacement of the scaffold during inside-out suturing of the scaffold to the meniscus.

The long-term results of meniscal scaffolds are less predictable. A loss of function of the meniscal scaffold has been reported during to fragmentation, shrinkage, and extrusion, which results in failure to increase articular cartilage coverage, to reduce peak pressure across the knee joint, and loss of a balanced load distribution across the knee.^8^ With regard to the method of implantation of the PGA scaffold coated with P(LA/CL) that we describe herein, preservation of the periphery of the meniscus is an essential indication for this surgery as, otherwise, the hoop tension is insufficient to support scaffold implantation. As such, additional surgical treatment for peripheral reconstruction of the meniscus might be needed in cases of meniscal extrusion. Although further research and clinical follow-up might identify further issues to be considered regarding meniscal scaffold implantation, based on our current evidence, appropriate surgical technique with adherence to strict indications might improve longer-term outcomes after scaffold implantation.

## Supplementary Data

ICMJE author disclosure forms

Video 1 (Slide 1) Degenerative meniscal tears are generally considered to be irreparable. In this video, we introduce a meniscal scaffold and describe the arthroscopic implementation technique for this scaffold. (Slide 2) This is a representative case of a meniscal complex tear, combining horizontal and flap tears at medial posterior horn of meniscus that had already undergone degenerative changes. A partial meniscectomy was performed in the degenerative area by using oval punch and shaver. After resection, the defect size was 14 mm of depth, and 16 mm of width. The edge of the remnant meniscus was considered sufficient to stimulate meniscal cell regeneration. The second step was scaffold preparation. A rein suture was set at the center of the scaffold. The size of the implanted scaffold was set to 1-2 mm over size of the defect, with a scalpel used to trim the scaffold as needed. The anteromedial portal was used to place the segmented scaffold at the appropriate intra-articular location using a grasper. The scaffold was then sutured, using an inside-out technique, to the adjacent native menisci. Suturing of the scaffold to the posterior horn is technically demanding. As such, we recommend that suture tension should, therefore, be regulated through the middle of the meniscus. The stability of the implanted scaffold was confirmed with probing and knee motion.
